# Effects of Body-Mass-Scaled Protein and Leucine Supplementation on Body Composition and Cardiometabolic Blood Markers During Weight Loss in Older Men—A Randomized Controlled Trial

**DOI:** 10.3390/nu18193255

**Published:** 2026-10-02

**Authors:** Benedict Wei Jun Pang, Yifan Yang, Bobby Kyungbeom Cheon

**Affiliations:** 1 Physical Education and Sports Science, National Institute of Education, Nanyang Technological University, Singapore 637616, Singapore; benedict.wj.pang@gmail.com; 2 Science of Learning in Education Centre, National Institute of Education, Nanyang Technological University, Singapore 637616, Singapore; 3 Eunice Kennedy Shriver National Institute of Child Health and Human Development, National Institutes of Health, Bethesda, MD 20892-2425, USA; bobby.cheon@nih.gov

**Keywords:** leucine, protein, alternate-day fasting, intermittent fasting, weight loss, muscle preservation, sarcopenia skeletal muscle, fat mass, HOMA-IR, insulin

## Abstract

**Background:** Overweight and obesity are burgeoning health epidemics. Diets including alternate-day fasting (ADF) promote weight loss but risk significant muscle loss, posing health implications to seniors at risk of sarcopenia. Leucine, the key muscle protein synthesis (MPS) driver, effectively stimulates MPS with lower calorie intake than protein. However, the effects of body-mass-scaled protein and leucine intakes are unclear. **Objectives:** To evaluate body-mass-scaled leucine or protein supplementation on body composition, sarcopenic, and cardiometabolic markers in older men during ADF. **Methods:** Sixty-one older men (BMI ≥ 23.0 kg/m^2^) underwent 28 days of ADF, randomized across three calorie-matched supplement groups: Control (C: 0.0 g/kg protein, 0.0 g/kg leucine), Leucine (L: 0.0 g/kg protein, 0.08 g/kg leucine), or Protein (P: 0.4 g/kg protein, 0.04 g/kg leucine), consuming three doses on feeding days and one on fasting days. Dual-energy X-ray absorptiometry, sarcopenic metrics, and blood markers (blood pressure, lipid panels, diabetic profiles) were measured. **Results:** There were no significant interactions (*p* > 0.05) in body composition, sarcopenic or health outcomes, except for diastolic blood pressure (*p* = 0.016) and HDL cholesterol (*p* = 0.013). There were significant main effects of time (*p* ≤ 0.05) with reductions in all body composition measures (excluding bone mineral density, *p* = 0.274), systolic blood pressure and lipid profiles (total and LDL cholesterol, total/HDL cholesterol ratio, triglycerides), and improvements in all muscle strength and functional sarcopenic outcomes. **Conclusions:** Dietary interventions alone that increase leucine and/or protein intake did not result in additional measurable improvements during this four-week intervention on body composition, sarcopenic, and cardiometabolic outcomes during weight loss.

## 1. Introduction

Overweight and obesity, the accumulation of excess body mass and fat mass, are a global public health epidemic recognized as a disease that requires clinical treatment [1]. In Singapore, overweight (body mass index, BMI 25.0–29.9 kg/m^2^) and obesity (BMI ≥ 30.0 kg/m^2^) are prevalent in 37.0% and 3.7% of older adults (≥60 years old), respectively [2]. These numbers increase to 46.0% and 16.3% when the BMI recommendations for Asian populations at increased risk (23.0–27.4 kg/m^2^) and high risk (≥27.5 kg/m^2^) of cardiometabolic diseases are used instead [3]. Given that excess body mass and fat mass increase the likelihood of developing several chronic conditions, such as metabolic syndrome, cardiovascular disease, diabetes, and cancer, as well as overall mortality [4,5], this increasing prevalence constitutes a growing public health concern.

However, older adults who are trying to lose weight face a two-pronged challenge. First, muscle mass and strength naturally decline at an increasing rate with age as part of the physiological aging process [6]. Second, while effective in reducing body mass [7,8] and fat mass [7,8], weight loss diets such as intermittent fasting, including alternate-day fasting (ADF; alternating between feeding and fasting days every 24 h), are typically accompanied by significant muscle loss [7,8]. Collectively, these factors exacerbate the loss of muscle mass and strength, especially when overweight or obese older adults try to lose weight through dieting alone. This leads to an increased risk of developing ‘sarcopenia’, a muscle disease associated with adverse health and functional outcomes [9]. Even more concerning is the condition known as ‘sarcopenic obesity’, the coexistence of sarcopenia and obesity, which exerts synergistically detrimental effects on cardiovascular and metabolic health [10]. Therefore, it is particularly important for older adults to preserve muscular outcomes when losing weight.

A high-protein diet of ≥1.20 g/kg (body mass)/day has been recommended as a measure to limit muscle loss during weight loss [11,12], due to its effects on stimulating muscle protein synthesis (MPS) and other anabolic pathways, including insulin secretion and the inhibition of muscle protein breakdown [12,13,14,15]. More recently, high leucine intake has been advocated for its pivotal role in activating MPS [16,17] to the same degree as high protein intake, but with lower overall protein consumption [18,19], potentially reducing overall caloric intake while maintaining or improving muscular outcomes. This is a particularly beneficial factor for older adults who may have decreased appetites or are trying to lose weight.

During four weeks of ADF in young adult men, we recently showed that supplementing with an additional 25 g of whey protein on fasting days did not lessen the weight-loss-induced muscle loss [20]. Leucine intakes of 0.02–0.08 g/kg (body mass)/meal over four weeks of ADF was also not effective in preserving muscle in young adult men (Preprint; manuscript in review) [21]. Nonetheless, the effects of body-mass-scaled protein and leucine supplementation, in addition to self-selected diets, on body composition, sarcopenic outcomes, and other cardiovascular and metabolic health indicators during weight loss remain particularly uncertain in older adults, who have been shown to require even more protein [22] and leucine [23] intake than young adults for similar MPS, due to an age-associated ‘anabolic resistance’ to protein and amino acid ingestion.

In animal models, leucine deficiency has been shown to worsen cognitive decline, while a high leucine diet improved cognitive function [24]. It is postulated that leucine deficiency inhibits mTORC1, thereby inducing neuronal autophagy, leading to cognitive decline. In human trials, 6 g/day of an amino acid mixture (containing 1.87 g of leucine) over 12 weeks improved cognition via the domains of attention and executive function in middle-aged and older adults [25]. Thus, the potential effect of leucine supplementation on cognition in our target population is of interest.

This study’s first objective was to investigate the effects of body-mass-scaled protein and/or leucine supplementation—in addition to self-selected diets that were tracked—on body composition and sarcopenic outcomes during four weeks of ADF-induced weight loss in older men. While we did not find a leucine dose–response in overall leucine intake among younger men [21], a supplementation intervention on top of self-selected diets in the present study was intended to result in much higher overall protein and leucine intakes. In addition, there is reason to believe that high leucine interventions have the potential to improve muscular outcomes in older adults, both at rest and in combination with resistance training [18,19]. Finally, the effects on cardiometabolic blood markers and cognitive function were examined. We hypothesized that increasing protein and leucine intake through supplementation would improve muscular, sarcopenic, health, and cognitive outcomes in older men during weight loss.

## 2. Materials and Methods

### 2.1. Study Design

**Figure 1:**
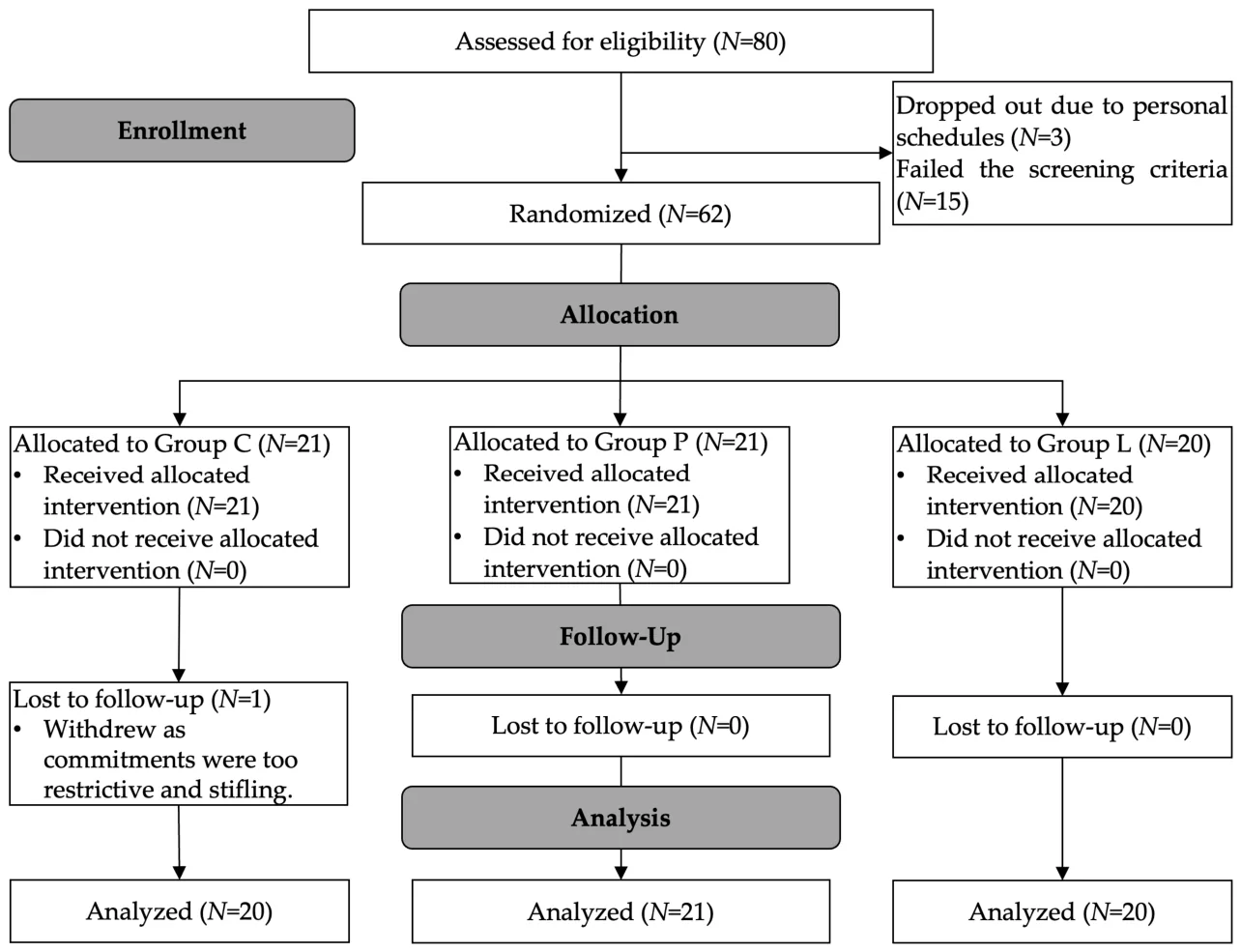
CONSORT participant flow diagram.

In this three-arm, parallel, double-blind randomized controlled trial, a total of 80 older men aged 60–89 years with a BMI ≥ 23.0 kg/m^2^ were recruited to undergo 28 days of ADF (Figure 1), where they alternated between days of feeding (ad libitum diet) and fasting (≤600 kcal consumed between 12 and 2 PM). Participants were recruited through newspaper advertisements, social media posts and word of mouth. Interested individuals were first contacted via messaging and/or voice call, where brief eligibility screening was performed before inviting them to the laboratory for in-depth screening. Participants were randomly assigned to one of three groups (1:1:1), where supplements were provided to be taken with each meal (i.e., three servings on each feeding day and one serving on each fasting day) on top of their self-selected diets: Leucine group (L), Protein group (P), and a Control group (C).

Participants were assigned using minimization with no random element to balance baseline body mass, BMI, age, and physical activity level. Allocation concealment was maintained by an independent investigator—uninvolved in participant recruitment or assessments—who manually executed the minimization protocol in Microsoft Excel to handle the multiple balancing factors, and the allocation sequence remained entirely concealed from and inaccessible to all other research staff and participants. Allocations were selected to minimize between-group differences in means and standard deviations of the balancing factors. Group assignments were disclosed solely to a supervisory staff member, who was responsible for assigning supplement packing duties without disclosing the participant identifiers. All other investigators, outcome assessors, supplement packers, and participants remained strictly blinded. This single-site trial was conducted at Nanyang Technological University (NTU) following approval by the NTU Institutional Review Board (IRB-2022-090, 9 June 2022). No changes were made to the methods or outcome measures after trial commencement (NCT07781410 with ClinicalTrials.gov, 17 August 2026; https://clinicaltrials.gov; retrospectively registered, as this study did not involve drug or medical device testing, was not classified as a clinical trial in Singapore, and was therefore not eligible for clinical trial registration in Singapore; https://www.hsa.gov.sg/other-regulations/clinical-trials/overview/, accessed on 28 August 2026).

### 2.2. Participant Screening

Prior to study enrollment, participants were screened according to the following inclusion criteria: (1) male, (2) ≥60 years old, (3) non-smoker (including other tobacco products such as shisha), and (4) BMI ≥ 23.0 kg/m^2^. Additionally, participants were excluded if they: (1) were unable to follow the physical activity and dietary requirements; (2) were vegetarians or vegans (protocol consideration: supplements provided contained dairy; practical considerations: vegans and vegetarians often require higher protein intakes due to the lower digestibility, bioavailability, and sub-optimal essential amino acid content of plant-based proteins compared to animal proteins [26]. Most importantly, on average, plant-based proteins naturally contain less leucine (~7.1%) than animal-based proteins (~8.1%) [27], which can systematically confound the findings of our study); (3) had known allergies or intolerance to dairy products (supplements provided may contain dairy); (4) had previously failed an exercise stress test; (5) had an existing medical condition that could be worsened by fasting; (6) had diabetes, pre-diabetes, high fasting blood glucose (>6.0 mmol/L), or uncontrolled hypertension (≥150/90 mmHg for ≥80 years old, ≥140/90 mmHg for <80 years old); (7) had liver or kidney conditions; (8) had a history of familial leucine-sensitive hypoglycemia (highly symptomatic hypoglycemia triggered by protein or leucine intake); (9) were on any long-term medications, including traditional Chinese medicine and other alternative medicine, drugs, steroids, or hormones that could affect any of the outcome measures in this study; (10) had existing musculoskeletal conditions that could be exacerbated by the physical assessments in this study; (11) had an electrical or metal implant within the body, including pacemakers or joint replacements; or (12) engaged in structured resistance training more than twice per week, or competed in resistance training-related competitions in the prior 6 months. All eligible participants provided written informed consent prior to their inclusion in the study.

### 2.3. ADF Protocol

The ADF program lasted continuously for four weeks (28 days), alternating every 24 h between feeding and fasting days, starting at 12 AM (midnight) each day. On fasting days, participants were allowed to consume a small, self-selected meal comprising ≤600 kcal that included one serving of the supplement provided, between 12 and 2 PM to standardize the total duration of fasting and feeding throughout the four weeks. A list of common food items and their respective caloric contents was provided as a guide, and unsweetened black coffee, black tea, and other zero-calorie beverages were permitted throughout the intervention duration to help them adhere to the fasting-day restrictions. On feeding days, participants were allowed ad libitum consumption of food and beverages with no restriction on the timing of meals throughout the 24 h period. Participants consumed one serving of the supplement provided for each of the three main meals for a total of three servings on feeding days.

Participants were randomly assigned to one of three groups according to the supplement assigned to them: Control group (C) received a supplement containing 0.0 g/kg protein and 0.0 g/kg leucine (Cadbury Drinking Chocolate), Protein group (P) received 0.4 g/kg protein and 0.04 g/kg leucine (Optimum Nutrition Gold Standard 100% Whey Protein), and Leucine group (L) received 0.0 g/kg protein and 0.08 g/kg leucine (Allmax Essentials Leucine Powder or Now Foods, Sports, L-Leucine Powder), which was masked using the same Cadbury Drinking Chocolate that was provided to group C, since pure leucine powder is odorless and flavorless. All three powdered supplements were calorie-matched per kg (body mass) and chocolate-flavored, packed in opaque, sealed pouches for blinding purposes.

Previously, the upper safety limit of leucine intake for older men was determined to be 0.50 g/kg/day [28]. Considering that the median older man in Singapore consumes about 90.8 g of protein per day [29], using 10% as a liberal estimate for leucine content [30], approximately 9.1 g/day or 0.13 g/kg/day (for a 70 kg individual) was derived. The highest leucine intake group, L, is expected to ingest 0.24 g/kg (0.12 g/kg for group P) of leucine on feeding days, equating to about 0.37 g/kg/day (0.25 g/kg/day for group P) of total leucine intake after accounting for their self-selected diets, which is within the upper safety limit for older adults. Concurrently, these values (0.25–0.37 g/kg/day) are well above the recommended dietary allowance (RDA; 0.081 g/kg/day) in older adults for MPS [23], and the protein provided from supplements (~1.2 g/kg/day) in group P, when combined with their diets, would far exceed the recommended ≥1.20 g/kg/day to limit muscle loss during weight loss [11,12].

### 2.4. Laboratory Sessions

**Figure 2:**
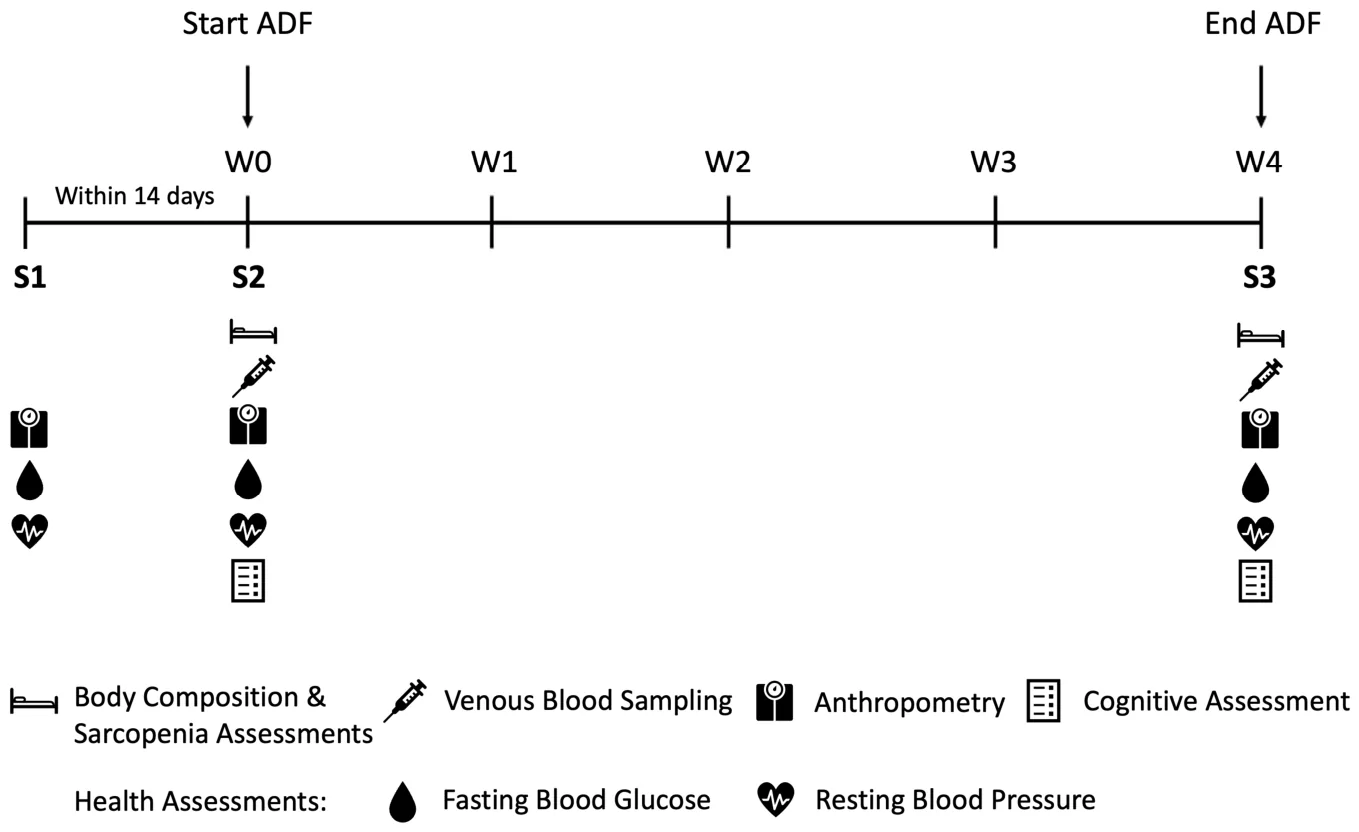
Schematic of laboratory sessions. ADF, Alternate-Day Fasting; W, Week; S, Session.

Over the five-week study period, participants attended three sessions at the Human Bioenergetics Laboratory (Physical Education and Sports Science, National Institute of Education, NTU, Singapore). Throughout the intervention, subjects logged and maintained daily physical activity, avoided starting new workout programs, and refrained from high-intensity exercise or resistance training. To minimize confounding variables, participants adhered to strict pre-test guidelines before each session, including visiting the laboratory at approximately the same time of day (±1 h) to account for diurnal variations, refraining from all intentional exercise and physical activity 48 h prior, abstaining from all alcoholic and caffeinated food and beverages 24 h prior, and observing an overnight fast of at least 10 h (plain water permitted) prior to each visit. The first laboratory session (Figure 2) was consent-taking and screening for eligibility (refer to Section 2.2), where participants also answered a physical activity questionnaire to determine their baseline weekly physical activity levels (details in Section 2.4.5). Afterward, participants were randomly allocated by minimization, and their supplementation was calculated and prepared based on their first laboratory-measured body mass. The second laboratory session, within two weeks after the first, was a baseline assessment session (i.e., anthropometry, body composition, sarcopenia assessments, health assessments, venous blood sampling, and cognitive function assessment; details in Section 2.4.1, Section 2.4.2, Section 2.4.3, Section 2.4.4, Section 2.4.5 and Section 2.4.6), and the start of the 4-week ADF protocol. Participants collected all the supplements for the entire 4-week ADF at this session. Participants returned to the laboratory for the third (final) session, on the same day of the week as their second (baseline) session, for post-intervention assessments identical to those performed at baseline.

#### 2.4.1. Anthropometry

Body height (m) and weight (kg) were measured on all three laboratory sessions using the seca 763 electronic weighing and measuring station (seca GmbH & Co. KG, Hamburg, Germany). Participants were dressed in comfortable, lightweight clothing (e.g., t-shirt and shorts) and measurements were taken with shoes off. BMI was calculated using weight divided by height squared (kg/m^2^).

#### 2.4.2. Body Composition

Body composition was assessed at the second and third laboratory sessions, corresponding to pre- and post-intervention, through dual-energy X-ray absorptiometry (DXA) scan using the Lunar Prodigy (GE Lunar Corporation Inc., Madison, WI, USA). Measurements including total and regional fat and lean mass (kg), total fat mass index (kg/m^2^), total and regional lean mass indexes (kg/m^2^), percentage body fat (%), bone mineral content (g), and bone mineral density (g/m^2^) were obtained.

#### 2.4.3. Sarcopenia Assessments

Participants were assessed for sarcopenia using the Asian Working Group for Sarcopenia (AWGS) 2019 criteria at the second and third laboratory sessions [31]. The outcome measures include (1) appendicular lean mass index (ALMI, kg/m^2^), a measure of skeletal muscle mass calculated as appendicular lean mass (lean mass of the four limbs) divided by height squared; (2) handgrip strength (HGS, kg), a measure of overall muscle strength; (3) gait speed test (GS); and (4) short physical performance battery (SPPB).

ALMI values were derived from the DXA scan results, where low muscle mass was defined as ALMI < 7.0 kg/m^2^ [31]. HGS was measured using the Jamar Plus Digital Hand Dynamometer (Patterson Medical, Cedarburg, WI, USA). Two trials were taken per arm in an alternating fashion with the participant in a seated position and arms held 90 degrees at the sides. The highest of four readings was used for analysis. Low muscle strength was defined as <28 kg [31]. Habitual GS was determined by a 6 m walk test. Participants started walking two meters before and stopped two meters after a demarcated 6 m distance to allow them to reach and then decelerate from their habitual gait speeds. Two trials were taken, and the average was used for analysis, with poor physical performance defined as <1.0 m/s [31].

The SPPB is a validated assessment for physical function [32]. A composite score (i.e., 0–12) was calculated for SPPB, which comprises three components: balance (i.e., ability to stand with feet in tandem, semi-tandem, and side-by-side for 10 s each), gait speed (i.e., faster time of two trials taken to walk 4 m), and repeated chair stands (i.e., time taken to complete five repetitions of sit-to-stand), with each component scored ordinally (i.e., 0–4). A total score of ≤9 indicates poor physical performance [31]. Alternatively, a repeated chair stands timing of ≥12 s could also be used to indicate poor physical performance.

Sarcopenia was defined and diagnosed according to AWGS 2019 criteria, where either low muscle strength or poor physical performance, in the presence of low muscle mass, constituted a sarcopenia diagnosis. Fulfilling all three criteria was an indication of severe sarcopenia [31].

#### 2.4.4. Health Assessments

Across all three sessions, fasting blood glucose (FG; mmol/L), resting blood pressure (mmHg) and resting heart rate (bpm) were assessed. FG was assessed from a capillary finger-prick blood sample using an ACCU-CHEK Guide glucometer and Safe-T-Pro Plus lancet (Roche Diabetes Care GmbH, Mannheim, Germany). Resting systolic blood pressure (SBP) and diastolic blood pressure (DBP), along with resting heart rate, were recorded using an OMRON HEM-7320 blood pressure monitor (OMRON Healthcare Co., Ltd., Kyoto, Japan).

#### 2.4.5. Venous Blood Sampling

On the second and third laboratory sessions, venous blood was drawn from an antecubital vein using a 22G needle into gold-top serum separator tubes. Tubes were inverted five times and kept at room temperature for 30 min. The samples were then centrifuged at 2000× *g* for 10 min at room temperature, utilizing slow acceleration and no braking. Innoquest Diagnostics Pte Ltd., Singapore. processed the samples on the day of collection to assess lipid profile, including cholesterol and triglycerides (mmol/L), and diabetic profile (insulin [mU/L], Homeostasis Model Assessment of Insulin Resistance [HOMA-IR], and glycated hemoglobin [HbA1c, %]).

#### 2.4.6. Physical Activity and Cognitive Assessment

The global physical activity questionnaire (GPAQ) [33] was completed at the first visit to assess baseline weekly physical activity level, expressed as metabolic equivalents (MET·min/week) derived from daily physical activity logs using standard GPAQ criteria. The Montreal Cognitive Assessment (MoCA) is a validated, sensitive tool for early detection of mild cognitive impairment, assessing the domains of visuospatial/executive function, naming, memory, attention, language, abstraction, delayed recall, and orientation [34]. It was administered on the second and third sessions.

#### 2.4.7. Daily and Weekly Online Logs

Throughout the 4-week ADF intervention, participants kept daily records of sleep patterns, supplement packet consumption, and physical activity in order to monitor adherence and account for potential lifestyle confounders. Physical activity level was quantified weekly using the Global Physical Activity Questionnaire (GPAQ) criteria. Participants also submitted four-day food logs each week (two feeding, two fasting days, each including one weekday and one weekend day) to determine total nutritional intake. Collectively, these records were used to verify protocol adherence and evaluate potential confounding effects of dietary intake, physical activity, and sleep behavior on intervention outcomes. The logs were completed online, allowing for close monitoring and timely reminders to log daily rather than rely on recall.

### 2.5. Sample Size Calculation

Lean mass was designated as the key primary outcome measure guiding the sample size calculation for this study, among the several pre-specified primary outcomes in our trial registration. Lean mass was prioritized for this purpose, given its relevance to muscle preservation during weight loss and the availability of a well-established effect size estimate from prior literature. Prior research reported a large effect size (Cohen’s f = 0.40) for lean mass retention when combining resistance training with variable protein intake during energy restriction [35]. However, because this trial isolates the dietary intervention without resistance training, a more conservative small-to-moderate effect size (f = 0.20–0.25) was assumed. An a priori power analysis using G*Power (version 3.1.9.7) for a mixed repeated-measures ANOVA (3 groups, α = 0.05, power = 0.80, correlation = 0.50) established a requirement of 14–22 participants per group. Factoring in a 10% attrition buffer, the total target sample size was set at 80 participants.

### 2.6. Statistical Analyses

Primary outcomes spanned two main domains: body composition (body mass, fat mass, lean mass, appendicular lean mass [ALM], BMI, fat mass index [FMI], lean mass index [LMI], ALM index [ALMI], percentage body fat [PBF], and trunk fat mass) and health biomarkers (SBP, DBP, FG, insulin, HOMA-IR, HbA1c, total cholesterol [TC], high-density lipoprotein cholesterol [HDL-C], low-density lipoprotein cholesterol [LDL-C], TC/HDL ratio, and triglycerides [TG]). Secondary outcomes included android and gynoid fat mass, bone mineral content (BMC) and density (BMD), sarcopenia metrics (ALMI, HGS, 6 m gait speed [6mGS], 5-times sit-to-stand [5STS], and the Short Physical Performance Battery [SPPB]), and cognitive function assessed via the Montreal Cognitive Assessment (MoCA). Analysis was performed on all available data from participants who completed the trial.

Data were analyzed using SPSS version 29.0 (Statistical Package for the Social Sciences, Chicago, IL, USA). Continuous variables are summarized as mean (SD) for descriptive spread—chosen due to variable normality across timepoints and complex distributions (the nonparametric tests are not tests of medians)—and as mean [95% CI] for inferential tests. Categorical data are reported as n (%). Pre- to post-intervention changes (Sessions 2 to 3) were evaluated using a 3 × 2 (Group × Time) Generalized Estimating Equation (GEE) with a robust estimator, exchangeable correlation matrix, and normal distribution with log or identity link function. Model selection prioritized optimal goodness-of-fit followed by lower parameter estimate covariance. Significant interaction effects were followed by simple effects testing with Sidak corrections for multiple comparisons, while main group effects were evaluated using Tukey’s post hoc test (homogeneous variances) or Games–Howell test (non-homogeneous variances). Baseline characteristics, potential confounders, and dietary intakes were compared using Welch ANOVA to handle unequal variance with equal group sample sizes. Significance level was set at *p* ≤ 0.05, with Cohen’s thresholds defining small (0.2), medium (0.5), and large (0.8) effect sizes.

## 3. Results

A total of *N* = 80 participants were recruited. After accounting for 19 dropouts (three withdrew before the start of the program due to personal schedules, 15 withdrew due to failing the screening criteria, and one withdrew after starting the program as commitments were too restrictive and stifling), a total of *N* = 61 participants completed the study and were included in the analyses. Participant characteristics are displayed in Table 1. Participant recruitment spanned November 2022 through May 2023, with data collection concluding in June 2023. Two participants reported light-headedness and weakness on the first fasting day of the program. One participant experienced mild stomach discomfort, bloating, constipation, and slight joint discomfort after consuming the supplements. Overall, participants tolerated the intervention well without any reported adverse events, rendering the planned non-systematic analysis of harms unnecessary. This aligns with expectations, as the maximum leucine dosage remained safely below established limits, and ADF protocols are widely documented as safe and well tolerated.

Compliance with the program was high. Overall compliance with the fasting-day meal timing (i.e., 12–2 PM) was 92.9 (10.4) %, with 93.4% of all participants having a compliance rate of 75% and above. In terms of fasting-day meal kcal (i.e., ≤600 kcal including supplement), overall compliance was moderate at 63.1 (28.6) %, with 52.5% of all participants having a compliance rate of 75% and above. For the 180 (out of 488 recorded) fasting-day meals that exceeded the limit, the average excess was 157.2 (150.5) kcal, ranging from 0.23 to 911.75 kcal. As for completion of supplements provided, the overall compliance was 98.6 (1.7) %, with 100% of all participants having a compliance rate of 75% and above.

### 3.1. Baseline Characteristics

Participants were minimized for baseline body mass, BMI, age, and physical activity level (Table 2). There was no significant difference between groups at baseline for body mass [F(2, 39) = 0.012, *p* = 0.988], BMI [F(2, 38) = 0.176, *p* = 0.839], age [F(2, 36) = 0.439, *p* = 0.648], or physical activity level [F(2, 38) = 0.231, *p* = 0.795].

### 3.2. Potential Confounders

Mean sleep duration and overall physical activity across the 4-week intervention are detailed in Table 3. There was no significant difference between groups for average sleep duration [F(2, 38) = 0.552, *p* = 0.581] and total physical activity [F(2, 35) = 0.578, *p* = 0.566].

### 3.3. Diet Analyses

Data for supplements given and consumed (i.e., given minus missed doses) are presented in Table 4. For groups C, P, and L, protein and leucine doses provided and consumed were in accordance with what was planned.

For supplements consumed, differences in energy (kcal/kg), protein (g/kg) and leucine (g/kg) per dose were analyzed. Energy intake was not significantly different between groups [F(2, 39) = 0.07, *p* = 0.933]. There were significant differences between groups for protein [F(2, 35) = 24,832.3, *p* < 0.001] and leucine [F(2, 26) = 61,104.6, *p* < 0.001]. Post hoc analyses showed significant differences (*p* < 0.001) in supplements consumed for protein and leucine for all pairwise comparisons, as intended.

Data for self-selected diet and diet consumed (i.e., self-selected diet plus supplements consumed) are presented in Table 5. Differences in energy (kcal/kg/day), protein (g/kg/day) and leucine (g/kg/day) intake were analyzed.

For self-selected diets, there were no significant differences between groups for feeding-day energy [F(2, 38) = 0.453, *p* = 0.639], protein [F(2, 38) = 0.315, *p* = 0.732], and leucine intakes [F(2, 38) = 0.464, *p* = 0.632]; fasting-day energy [F(2, 38) = 1.522, *p* = 0.231], protein [F(2, 38) = 0.156, *p* = 0.856], and leucine intakes [F(2, 38) = 0.084, *p* = 0.92]; or overall average energy [F(2, 38) = 0.754, *p* = 0.477], protein [F(2, 38) = 0.168, *p* = 0.846], and leucine intakes [F(2, 38) = 0.31, *p* = 0.735].

For diet consumed (including supplements), feeding-day energy intake did not differ significantly between groups [F(2, 38) = 0.455, *p* = 0.638], but feeding day protein [F(2, 38) = 103.064, *p* < 0.001] and leucine [F(2, 37) = 558.889, *p* < 0.001] intakes did. Post hoc analyses showed group P differed significantly from groups C and L in protein intake (*p* < 0.001), and all pairwise comparisons differed significantly in leucine intake (*p* < 0.001).

Similarly, fasting-day energy intake did not differ significantly between groups [F(2, 38) = 1.526, *p* = 0.23], but fasting-day protein [F(2, 38) = 71.994, *p* < 0.001] and leucine [F(2, 38) = 288.309, *p* < 0.001] intakes did. Post hoc analyses showed group P differed significantly from groups C and L in protein intake (*p* < 0.001), and all pairwise comparisons differed significantly in leucine intake (*p* < 0.001).

Overall energy intake did not differ significantly between groups [F(2, 38) = 0.758, *p* = 0.476], but overall protein [F(2, 38) = 113.876, *p* < 0.001] and leucine [F(2, 38) = 602.201, *p* < 0.001] intakes did. Post hoc analyses showed group P differed significantly from groups C and L in protein intake (*p* < 0.001), and all pairwise comparisons differed significantly in leucine intake (*p* < 0.001).

The results confirmed successful implementation of the dietary intervention, with group L consuming more leucine than group P, and group P more than group C, while group P consumed ~double the protein of the other groups, with no difference in energy intake among the groups.

### 3.4. Body Composition

**Figure 3:**
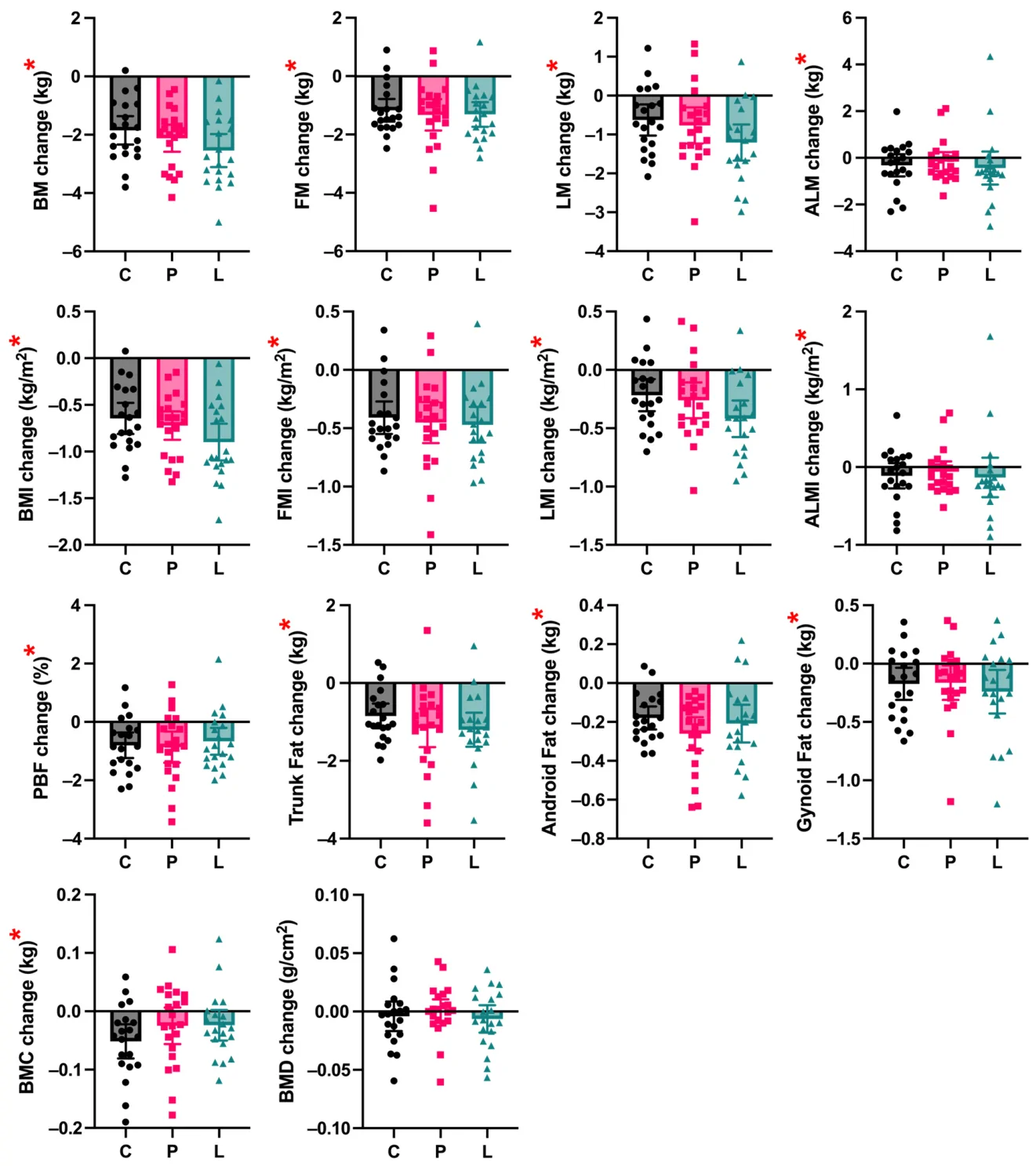
Changes in body composition (post minus pre). Bar chart presented as mean and 95% CI, with scatterplot of individual data points. C, Control group; P, Protein group; L, Leucine group; BM, body mass; FM, fat mass; LM, lean mass; ALM, appendicular lean mass; BMI, body mass index; FMI, fat mass index; LMI, lean mass index; ALMI, appendicular lean mass index; PBF, percentage body fat; BMC, bone mineral content; BMD, bone mineral density. * Significant main effect of time.

There were no significant time x group interaction effects for any body composition measure (*p* = 0.102–0.825). Main effects of time were significant (*p* < 0.05; Table 6) with a decrease post-intervention for all measures, except bone mineral density (BMD; *p* = 0.274). Figure 3 displays the pre–post changes in body composition for each group. Main effects of group (*p* = 0.086–0.999) were not significant for any measure.

### 3.5. Sarcopenic Markers

Sarcopenic measures are presented in Table 7. Pre- and post-intervention, respectively, the overall prevalence across all groups for low muscle mass was 6.6% and 13.1%, low muscle strength 14.8% and 13.1%, poor physical performance 8.2% and 3.3%, and sarcopenia/severe sarcopenia 4.9% and 6.6%.

McNemar’s (two proportions) tests showed no significant pre- to post-intervention change in the proportion of individuals with low muscle mass (χ^2^ = 2.67, *p* = 0.103), low muscle strength (χ^2^ = 0.33, *p* = 0.564), poor physical performance (χ^2^ = 1.80, *p* = 0.180), or sarcopenia (χ^2^ = 1.00, *p* = 0.317).

There were no significant interaction effects for any of the sarcopenic measures (*p* = 0.066–0.795). However, main effects of time were significant for all measures, with post-intervention changes in ALMI (−0.10 kg/m^2^, 95% CI [−0.20, −0.00], *p* = 0.047, d = −0.12), HGS (+1.44 kg, 95% CI [0.66, 2.22], *p* < 0.001, d = 0.21), 6mGS (+0.05 m/s, 95% CI [0.02, 0.08], *p* < 0.001, d = 0.38), 5STS (−0.92 s, 95% CI [−1.23, −0.60], *p* < 0.001, d = −0.61), and SPPB score (+0.08, 95% CI [0.00, 0.16], *p* = 0.05, d = 0.35). Main effects of group were not significant for any measure (*p* = 0.061–0.980).

### 3.6. Health Markers and Cognitive Function

Health characteristics and cognitive function are presented in Table 8. Due to unsuccessful venipuncture, venous blood samples were missing for one (pre) and three (post) participants from the control group, and one (pre) participant from the leucine group. Analysis of missing data was handled through GEE. There were no significant interaction effects for any health measure (*p* = 0.111–0.909), except for DBP (*p* = 0.016) and HDL cholesterol (*p* = 0.013), while SBP trended towards a significant interaction (*p* = 0.061). Simple effects of time (pre–post) were tested at different levels of group for DBP and HDL cholesterol. For DBP, pairwise comparisons showed that only groups P (−5.29 mmHg, 95% CI [−7.97, −2.61], *p* < 0.001, d = −0.98) and L (−4.30 mmHg, 95% CI [−6.98, −1.62], *p* = 0.002, d = −0.59) had a significant decrease post-intervention. The magnitude of change was similar between groups P and L (*p* = 0.943). As for HDL cholesterol, pairwise comparisons with Sidak corrections showed a significant decrease post-intervention in group P (−0.07 mmol/L, 95% CI [−0.11, −0.02], *p* = 0.005, d = −0.34).

Main effects of time were significant for the following variables, with significant improvement post-intervention in SBP (−5.39 mmHg, 95% CI [−7.57, −3.22], *p* < 0.001, d = −0.53), TC (−0.23 mmol/L, 95% CI [−0.39, −0.06], *p* = 0.007, d = −0.26), LDL-C (−0.16 mmol/L, 95% CI [−0.31, −0.01], *p* = 0.04, d = −0.21), TC/HDL ratio (−0.15, 95% CI [−0.30, −0.01], *p* = 0.035, d = −0.18), TG (−0.15 mmol/L, 95% CI [−0.24, −0.05], *p* = 0.002, d = −0.27), and MoCA scores (+0.8, 95% CI [0.3, 1.31], *p* = 0.002, d = 0.38). Main effects of group were not significant for these measures (*p* = 0.109–0.874). Main effects of time (*p* = 0.120–0.275) and group (*p* = 0.696–0.941) were not significant for FG, insulin, HOMA-IR, and HbA1c.

## 4. Discussion

In this study, the effectiveness of body-mass-adjusted protein and leucine supplementation on body composition, sarcopenic outcome measures, cardiometabolic blood markers, and cognitive function during short-term ADF-induced weight loss in older men was investigated. To account for the contributions and confounding effects of physical activity and sleep, daily logs were maintained by the participants and analyzed. Participants were also assigned to groups using a minimization process for body mass, BMI, age, and physical activity level to minimize baseline differences. Most importantly, there were no significant differences between groups for self-selected (i.e., excluding the supplements) body-mass-adjusted energy, protein, or leucine intakes, and the leucine and protein supplemental doses provided, even after accounting for missed doses, were in accordance with the study design.

Similar to the findings of our previous study in young adult men [21], there were significant decreases in all measures of body composition post-intervention except for BMD. There was a significant loss in body mass of 2.2 kg (2.8%) over four weeks, which equates to about 0.5 kg/week, and among the 61 participants, only one participant gained weight (control group). This is a clinically significant finding, as it shows that a short-term ADF protocol is effective in inducing meaningful reductions in body mass among normal-weight to obese (BMI ≥ 23.0 kg/m^2^) older (≥60 y) men. Notably, the participants in this study lost more fat mass than lean mass. On average, fat mass decreased by 1.3 kg (58.5%) while lean mass decreased by 0.9 kg (40.0%). This is in line with current literature, where a loss in lean mass of between 25 and 42% of total weight loss can be expected regardless of the type of weight-loss diet [7,36,37]. Interestingly, 11 of the 61 participants gained lean mass post-intervention (five, four, and two from the control, protein, and leucine groups, respectively) while losing weight.

The finding that there were no significant interaction effects for any body composition measure was unexpected. Previously, two meta-analyses including young and older adults reported that high protein intake during calorie restriction with no concurrent exercise training led to significantly greater post-intervention reductions in fat and body mass, and significantly less muscle loss, compared to the control [38,39]. The meta-analysis by Wycherley et al. [39] included participants with a mean age of 20–62 y, and the average protein intake for the protein group was 1.25 g/kg/day vs. 0.72 g/kg/day in the control group, while the meta-analysis by Kim et al. [38] included older adults with a mean age of 50–62 y, and high vs. low protein was defined as ≥1.0 g/kg/day vs. <1.0 g/kg/day. In the present study, group P consumed 1.36 g/kg/day vs. 0.66 g/kg/day in group C, indicating that group P surpassed the 1.0–1.25 g/kg/day intake range reported in these meta-analyses, and with a greater between-group difference in protein intake than those reported in the meta-analyses. In fact, the protein intake of group P was well in excess of the ≥1.20 g/kg/day that is recommended during weight loss [40] and in older adults [41]. Similarly, the leucine intake for groups P (0.12 g/kg/day) and L (0.21 g/kg/day) exceeded the recommended daily intakes in older adults (0.081 g/kg/day) [23], and the supplemental leucine doses consumed (approximately 3.2 g and 6.3 g for P and L, respectively) met or exceeded the 1.9–4.2 g known to maximize MPS in one sitting [42,43,44,45].

Notably, the lack of interactions could be partially attributed to the ‘anabolic resistance’ experienced by the older adults in the present study (mean age 65 y), in contrast to the younger participant groups included in the meta-analyses by Kim et al. [38] and Wycherley et al. [39], where higher protein and leucine doses are required to optimize MPS [16,46,47,48]. While the study design did cater for this greater requirement, the undernutrition experienced on fasting days could have further contributed to the lack of a significant finding. The fasting-day protein intakes of 0.67 and 0.30 g/kg/day for groups P and L, respectively, were well below the RDA of 0.8 g/kg/day [49]. Group P also consumed 0.06 g/kg/day leucine on fasting days, below the RDA of 0.081 g/kg/day for older adults [23]. While 3 g of leucine has been shown to increase MPS to the same extent in high (25 g whey) and low (10 g milk) protein doses both at rest and post-resistance training in older adults (older women, mean age 69 y) [19], the MPS-stimulatory effect of leucine intake is transient and promptly reverts to baseline levels within just 2 to 3 h [42,43,44,45]. Consequently, maintaining elevated MPS may require repeated protein and leucine intakes [50], which could not be achieved on fasting days using the ADF protocol.

Incidentally, while increased leucine intakes through leucine supplementation over longer periods (up to three months) significantly increased MPS in older adults [51], this failed to increase lean mass compared to a control. Similarly, long-term protein intakes over 5 years were not associated with muscle cross-sectional area changes in older adults (70–79 y) [52]. Thus, going beyond a nutrition-only approach, a more crucial factor that could explain the statistical indifference in findings could be the lack of a structured resistance training protocol. Indeed, among middle- to older-aged women, a high-protein, high-leucine intervention significantly improved body composition only when combined with a structured exercise program [53]. A systematic review and meta-analysis further showed that higher protein intake combined with resistance training led to greater ALM and HGS in older adults than lower protein controls, while increased protein intake alone had no incremental benefit on muscle mass or strength [54]. Put together, the muscle-preserving impact that nutritional interventions alone (i.e., increased protein or leucine intakes) exert may be insufficient to improve muscular outcomes, and it may be prudent to combine such nutritional interventions with structured resistance training to elicit a significant and pronounced effect.

Interestingly, significant improvements in the physical components of sarcopenia (no interaction effects), including muscle strength and physical performance, were observed in the present study. Previously, it has been shown that diet-induced weight loss, either alone or combined with exercise, significantly improves physical function [55], likely through reducing inflammatory markers associated with physical decline [56]. Recently, leucine supplementation twice a day (3 g/dose; 0.04 g/kg/dose for a 70 kg individual) over 13 weeks was also found to preserve muscle mass and physical function compared to a placebo-controlled group [57]. However, participants in that study were nursing home residents with an average age of 79 y, compared to healthy community-dwelling adults aged ~65 y recruited in this study. In the present study, the average scores of muscle strength and physical function were well above the sarcopenia diagnosis cut-offs, indicating that our target population could have already been too robust at baseline for the differential protein and leucine intakes to make a further difference. As pointed out by the authors of previous systematic reviews [51,58], there is a likelihood that the benefits associated with leucine supplementation may only truly stand out in populations with significant physical and/or physiological decline, such as the older old (≥75 y), malnourished, or those already diagnosed with sarcopenia, osteoporosis, and other comorbidities. Separately, structured resistance training (3 x/week), with or without high-protein, high-leucine supplementation, during a diet-induced weight loss program, has been shown to improve HGS significantly [59], further illustrating that resistance training may indeed be the key factor in preserving muscular outcomes during weight loss and aging.

While there was a significant but small (−33.4 g, d = −0.1) loss in BMC, there were no significant changes in BMD, the variable of interest in determining osteopenia and osteoporosis. Thus, the results suggest that this short-term ADF protocol did not adversely affect bone health even among older adults (≥60 y), which agrees with the previous literature on ADF of 6 weeks to longer-term studies of more than 6 months in duration [60,61,62], and the findings of our previous study [21].

There was a significant reduction in SBP, and a significant interaction effect for DBP, where only groups P and L had a significant decrease in DBP. SBP also trended towards a significant interaction (*p* = 0.061), with pairwise comparisons yielding a similar conclusion as DBP (i.e., significant reductions only for groups P and L). The ability of leucine to reduce blood pressure has been suggested previously, at least indirectly, by its effects on promoting insulin and glucose homeostasis [63,64], and the stimulation of MPS for muscle building to increase metabolism and fat loss, in turn lowering blood pressure [64]. On the other hand, TG, LDL-C, TC, and TC/HDL-C ratio decreased post-ADF regardless of the group, indicating that there were significant improvements in cardiovascular markers during the ADF intervention regardless of protein or leucine intake. The benefits of diet and weight loss alone could have resulted in these improvements and overshadowed any additional benefits from increased protein and leucine intakes [65,66,67].

There were no improvements to fasting glucose, HbA1c, fasting insulin and HOMA-IR post-intervention, indicating that the ADF protocol did not benefit diabetic health regardless of protein or leucine intake. This is consistent with previous meta-analyses across cohorts with and without diabetes that demonstrate no significant improvements in diabetic profiles [7,68,69,70,71], except for one study reporting minor decreases in fasting glucose and HOMA-IR [72]. Although leucine has been reported to stabilize blood glucose [53] and enhance lipid metabolism [73], such effects may be masked by the confounding impact of concurrent fasting and weight loss [74,75]. Furthermore, given that carbohydrates (i.e., the masking agent, Cadbury Drinking Chocolate), whey protein and leucine are highly insulinotropic, and as a result have sometimes been linked to insulin resistance and diabetes [15,76,77], it is possible that the high amounts of these supplements provided (56 servings over four weeks) in addition to their self-selected diets negated the beneficial impact of fasting and weight loss on diabetic health, which could explain the contrast in findings with our previous study, where ADF in combination with 0.04–0.16 g/kg/day leucine intake improved diabetic profiles [21]. Most importantly, diabetic health did not deteriorate post-intervention in any group.

Cognitive test scores significantly improved post-intervention with no interaction effect. In RCTs and systematic reviews, weight loss significantly improved cognitive scores, especially in the domains of memory, attention, and executive function [78,79], with the proposed mechanisms being improved insulin sensitivity—however, it should be noted that fasting insulin, HOMA-IR, and other diabetic profiles did not change in the present study—and decreased inflammation, which have been shown to enhance neuroplasticity and neuro-facilitation of the brain in animal models, leading to better cognitive function [78,79]. High protein intakes have also been observed to be significantly associated with better cognitive functions [80], while leucine deficiency was associated with greater cognitive impairment in animal studies [24]; however, interaction effects were not present in this study. Notably, the MoCA cognitive test was developed to be a sensitive tool for detection of early mild cognitive impairment, i.e., MoCA score ≤ 25 [34], and studies on its normative data for healthy individuals are scarce [81]. Thus, it might not be as sensitive a tool for the robust older adults in this study, which could explain the lack of an interaction effect. Nonetheless, the exact mechanisms linking weight loss, dietary protein, and leucine intakes to cognitive function warrant further investigation.

As an extension to our previous study performed on young men [21], the main limitation of recruiting only older men for this study is that the findings may not be generalizable to older women, given sex-specific physiological differences and the potentially greater anabolic resistance experienced by postmenopausal women compared to older men [82]. Although physical activity can confound muscle anabolism, activity levels were not strictly controlled because enforcing simultaneous diet and exercise restrictions could be too restrictive and affect adherence. Instead, physical activity was accounted for and analyzed through daily physical activity logs, and participants were instructed maintain consistent activity, refrain from resistance training, and avoid initiating new exercise programs during the intervention. Dietary intake was also not controlled—rather, participants received caloric guidance for common local foods on fasting days and logged their food intake four days per week for subsequent analysis. Compliance with the fasting-day energy intake was modest (63.1%), which could affect the uniformity of the ADF intervention and influence between-group comparisons. However, the average excess was only about 157.2 (150.5) kcal, and most meals (308/488) complied with the guidelines, resulting in an overall calorie deficit that led to weight loss across all groups. More importantly, protein and leucine content were as intended, and given the consistency in the lack of interaction effects across variables, fasting-day energy intake was unlikely to have confounded the study outcomes. While it was challenging to ensure compliance with the diet and physical activity restrictions, the research team provided daily reminders to the participants and ensured that the logs were maintained daily and kept up to date. Clinical trial registration was obtained retrospectively in August 2026 (participant recruitment began in November 2022; data collection concluded in May 2023); however, it should be noted that no changes were made to the methods or outcome measures after trial commencement, and all primary and secondary outcomes reported in the manuscript had been defined before analyses. In addition, minimization was employed to reduce experimental bias and baseline between-group differences, alongside strict protocols including allocation concealment, double-blinding, and standardized procedures prior to each laboratory session. Given these safeguards, and that the study yielded predominantly null findings, retrospective registration is unlikely to have biased the reported outcomes. As all three groups underwent ADF without a non-ADF control group, the main effects of time reported in this study cannot be definitively attributed to ADF as a causal and independent contributor. Finally, a notable limitation of the ADF protocol is that fasting-day intakes of protein (groups P and L) and leucine (group P) were insufficient to preserve muscle mass during weight loss, despite appropriate per-meal dosing design.

## 5. Conclusions

This is the first study to investigate the effects of leucine and protein supplementation scaled to body mass during short-term weight loss in older adults. Existing studies often do not compare different amounts of leucine supplementation, and those that do, do not scale the dose to individual body mass due to the extensive logistics and manpower required. This study focused on supplementation in addition to habitual food intake, making the findings more translatable and applicable to real-world conditions.

High leucine (0.08 g/kg/serving), or moderate leucine (0.04 g/kg/serving) in combination with high protein (0.4 g/kg/serving), did not result in additional measurable improvements in body composition, sarcopenic outcomes, cardiometabolic health, or cognitive function compared to a control group during weight loss. On the other hand, ADF was safe, tolerable, and well-adhered to in this cohort, and was associated with improvements in physical function, cognitive performance, and cardiovascular markers even among older men. At present, the position stand from the International Society of Sports Nutrition (ISSN) and current consensus indicate that high protein intake (1.6 g/kg/day) combined with resistance training is the best way to preserve muscle during weight loss [11,12]. When not undergoing weight loss, resistance training outweighs high protein intake alone in improving muscle mass and strength, with the combined effects of concurrent resistance training and high protein intake being greater than either in isolation [54,83]. Together with the findings of this study and our previous studies [20,21], the evidence suggests that the anabolic stimulus from resistance training may be key to improving muscular and sarcopenic outcomes in older adults, especially during weight loss, which may be further enhanced by increased protein and leucine consumption.

## Figures and Tables

**Table 1:** Participant characteristics.

	Group	Control		Protein		Leucine	
	Session	Pre	Post	Pre	Post	Pre	Post
	*N*	20	20	21	21	20	20
**Age (y)**	Mean	64	64	65	65	65	65
	SD	3	3	4	4	7	7
	Min	60	60	60	60	60	60
	Max	70	71	74	74	89	89
**Height (m)**	Mean	1.69	1.69	1.71	1.71	1.68	1.68
	SD	0.03	0.03	0.05	0.05	0.08	0.08
	Min	1.63	1.63	1.61	1.61	1.51	1.51
	Max	1.74	1.74	1.79	1.79	1.82	1.82
**Body Mass (kg)**	Mean	78.2	76.4	79.2	77.1	78.6	76.0
	SD	11.3	11.1	12.3	12.2	12.4	12.4
	Min	62.9	61.3	63.7	62.6	58.5	56.0
	Max	120.8	118.0	109.8	106.5	104.6	100.9
**Fat Mass (kg)**	Mean	25.3	24.1	25.4	24.0	25.3	24.0
	SD	6.7	6.8	8.7	8.5	6.2	6.0
	Min	15.0	13.8	12.7	11.3	11.6	12.7
	Max	48.4	47.0	46.0	45.0	40.2	39.2
**Lean Mass (kg)**	Mean	49.9	49.3	50.7	50.0	50.5	49.3
	SD	5.4	5.2	4.9	5.1	7.6	7.5
	Min	42.3	41.6	44.4	42.9	36.3	34.5
	Max	68.5	67.3	60.6	61.9	68.3	66.6
**Appendicular Lean Mass (kg)**	Mean	22.6	22.3	23.0	22.8	22.6	22.2
	SD	2.1	2.5	2.7	2.9	4.4	4.0
	Min	17.9	18.1	19.4	19.1	14.7	14.6
	Max	27.3	29.3	27.6	28.6	33.6	30.6
**Body Mass Index (kg/m^2^)**	Mean	27.5	26.8	27.0	26.3	27.7	26.8
	SD	3.5	3.5	3.4	3.4	3.8	3.9
	Min	23.0	22.4	22.9	22.3	23.0	22.0
	Max	40.6	39.7	34.3	33.4	38.9	38.9
**Fat Mass Index (kg/m^2^)**	Mean	8.9	8.5	8.6	8.2	9.0	8.5
	SD	2.2	2.3	2.7	2.7	2.3	2.2
	Min	5.5	5.0	4.3	3.8	3.9	4.3
	Max	16.3	15.8	15.2	14.9	15.5	15.1
**Lean Mass Index (kg/m^2^)**	Mean	17.5	17.3	17.3	17.1	17.8	17.4
	SD	1.6	1.6	1.1	1.3	1.9	1.9
	Min	15.8	15.8	15.6	15.0	14.4	13.7
	Max	23.0	22.6	19.3	19.3	22.5	22.8
**Appendicular Lean Mass Index (kg/m^2^)**	Mean	7.9	7.8	7.9	7.8	7.9	7.8
	SD	0.6	0.8	0.7	0.8	1.0	1.1
	Min	6.8	6.9	6.9	6.7	5.9	5.8
	Max	9.2	9.9	9.3	9.0	10.2	10.6
**Percentage Body Fat (%)**	Mean	32.0	31.2	31.3	30.5	31.9	31.3
	SD	4.2	4.5	6.1	6.4	4.8	4.4
	Min	23.9	22.5	18.6	16.9	17.1	19.2
	Max	40.1	39.8	44.7	44.6	39.9	39.0
**Trunk Fat Mass (kg)**	Mean	15.7	14.9	16.3	15.2	15.8	14.6
	SD	4.5	4.4	5.5	5.3	4.2	3.6
	Min	8.2	7.5	8.2	7.0	6.7	7.7
	Max	31.5	29.5	30.1	26.9	25.9	22.4
**Android Fat Mass (kg)**	Mean	2.9	2.7	3.1	2.8	3.0	2.8
	SD	0.7	0.8	1.0	1.0	0.8	0.8
	Min	1.8	1.5	1.5	1.3	1.4	1.5
	Max	5.4	5.2	5.7	5.2	5.2	4.8
**Gynoid Fat Mass (kg)**	Mean	3.9	3.7	3.8	3.6	3.9	3.6
	SD	1.2	1.1	1.4	1.3	0.9	0.8
	Min	2.6	2.7	2.4	2.0	2.4	2.4
	Max	8.0	7.4	7.3	7.1	5.1	5.4
**Bone Mineral Content (kg)**	Mean	3.03	2.97	3.08	3.05	2.84	2.82
	SD	0.44	0.43	0.38	0.37	0.49	0.50
	Min	2.42	2.32	2.60	2.53	2.28	2.23
	Max	3.97	3.95	4.26	4.11	3.90	3.92
**Bone Mineral Density (g/cm^2^)**	Mean	1.23	1.22	1.23	1.23	1.18	1.17
	SD	0.11	0.10	0.08	0.08	0.09	0.09
	Min	1.11	1.07	1.11	1.10	1.04	1.05
	Max	1.48	1.42	1.47	1.41	1.37	1.38

**Table 2:** Baseline characteristics.

	Group	*N*	Mean	SD	Min	Max
**Body Mass (kg)**	**C**	20	78.6	11.4	63.5	121.9
	**P**	21	79.2	12.3	63.9	110.8
	**L**	20	78.9	12.4	58.0	104.0
**BMI (kg/m^2^)**	**C**	20	27.6	3.6	23.3	41.2
	**P**	21	27.1	3.4	23.0	34.7
	**L**	20	27.7	3.7	23.1	39.0
**Age (y)**	**C**	20	63.8	3.2	60.0	70.0
	**P**	21	64.8	4.2	60.0	74.0
	**L**	20	65.0	7.3	60.0	89.0
**GPAQ Physical** **Activity Level (MET·min)**	**C**	20	1752	1233	40	4320
	**P**	21	1771	1899	0	7680
	**L**	20	1516	1202	0	3600

C, Control group; P, Protein group; L, Leucine group; BMI, Body Mass Index; GPAQ, Global Physical Activity Questionnaire; MET, metabolic equivalent of task.

**Table 3:** Potential Confounders during the 4-Week Intervention.

	Group	*N*	Mean	SD	Min	Max
**Average Sleep (h/day)**	**C**	20	7.2	1.1	5.5	10.5
	**P**	21	7.5	0.8	5.8	9.2
	**L**	20	7.3	0.9	5.0	9.2
**Total Physical Activity (MET·min)**	**C**	20	4882	2362	1020	9628
	**P**	21	5834	4642	540	18,840
	**L**	20	4361	4173	0	18,840

C, Control group; P, Protein group; L, Leucine group; MET, metabolic equivalent of task.

**Table 4:** Supplement analyses per meal.

Group	*N*	Energy (kcal)	Protein (g)	Leucine (g)	Fat (g)	CHO (g)	Protein (%)	Fat (%)	CHO (%)	Energy (kcal/kg)	Protein (g/kg)	Leucine (g/kg)
*Given*												
**C**	20	149 (22)	2 (0)	0.1 (0.0)	2 (0)	28 (4)	6 (0)	13 (0)	76 (0)	1.90 (0.00)	0.03 (0.00)	0.00 (0.00)
**P**	21	150 (23)	30 (5)	3.2 (0.5)	1 (0)	4 (1)	80 (0)	8 (0)	10 (0)	1.90 (0.00)	0.38 (0.00)	0.04 (0.00)
**L**	20	149 (24)	2 (0)	6.4 (1.0)	2 (0)	23 (4)	5 (0)	10 (0)	63 (0)	1.90 (0.00)	0.02 (0.00)	0.08 (0.00)
*Consumed*												
**C**	20	147 (22)	2 (0)	0.1 (0.0)	2 (0)	28 (4)	6 (0)	13 (0)	76 (0)	1.87 (0.03)	0.03 (0.00)	0.00 (0.00)
**P**	21	148 (23)	30 (5)	3.1 (0.5)	1 (0)	4 (1)	80 (0)	8 (0)	10 (0)	1.87 (0.04)	0.37 (0.01)	0.04 (0.00)
**L**	20	147 (23)	2 (0)	6.3 (1.0)	2 (0)	23 (4)	5 (0)	10 (0)	63 (0)	1.87 (0.03)	0.02 (0.00)	0.08 (0.00)

Data presented as mean (SD). C, Control group; P, Protein group; L, Leucine group; CHO, Carbohydrates.

**Table 5:** Diet analyses.

Group	*N*	Energy (kcal)	Protein (g)	Leucine (g)	Fat (g)	CHO (g)	Protein (%)	Fat (%)	CHO (%)	Energy (kcal/kg)	Protein (g/kg)	Leucine (g/kg)
*Self-selected (Feeding Day)*												
**C**	20	1536 (324)	70 (16)	5.2 (1.2)	58 (14)	180 (44)	18 (2)	34 (5)	47 (5)	19.88 (4.80)	0.91 (0.24)	0.07 (0.02)
**P**	21	1510 (366)	73 (18)	5.4 (1.4)	63 (18)	160 (42)	19 (3)	38 (4)	42 (6)	19.43 (5.44)	0.93 (0.26)	0.07 (0.02)
**L**	20	1398 (422)	75 (23)	5.7 (1.7)	54 (19)	149 (51)	22 (4)	34 (6)	43 (8)	18.20 (6.23)	0.99 (0.36)	0.07 (0.03)
*Self-selected (Fasting Day)*												
**C**	20	427 (146)	22 (9)	1.6 (0.7)	15 (6)	50 (20)	19 (6)	31 (9)	45 (14)	5.57 (2.03)	0.29 (0.13)	0.02 (0.01)
**P**	21	449 (113)	23 (7)	1.7 (0.6)	17 (5)	51 (15)	21 (5)	34 (6)	45 (9)	5.84 (1.79)	0.30 (0.10)	0.02 (0.01)
**L**	20	364 (134)	21 (9)	1.7 (0.8)	13 (6)	40 (18)	22 (8)	31 (11)	41 (13)	4.77 (2.12)	0.28 (0.13)	0.02 (0.01)
*Self-selected (Overall)*												
**C**	20	982 (212)	46 (11)	3.4 (0.9)	37 (9)	115 (29)	19 (3)	34 (4)	47 (5)	12.72 (3.18)	0.60 (0.17)	0.04 (0.01)
**P**	21	980 (204)	48 (11)	3.6 (0.8)	40 (10)	105 (25)	20 (3)	37 (4)	43 (6)	12.64 (3.26)	0.62 (0.16)	0.05 (0.01)
**L**	20	881 (235)	48 (13)	3.7 (1.0)	34 (11)	94 (29)	22 (4)	34 (6)	43 (8)	11.49 (3.68)	0.63 (0.21)	0.05 (0.02)
*Consumed (Feeding Day)*												
**C**	20	1976 (331)	77 (16)	5.6 (1.2)	64 (14)	263 (48)	16 (2)	29 (4)	53 (4)	25.48 (4.79)	1.00 (0.24)	0.07 (0.02)
**P**	21	1955 (375)	162 (24)	14.8 (2.2)	67 (18)	171 (42)	34 (4)	30 (3)	35 (5)	25.05 (5.45)	2.06 (0.26)	0.19 (0.02)
**L**	20	1840 (420)	81 (22)	24.6 (3.0)	59 (19)	219 (53)	18 (3)	29 (5)	48 (6)	23.81 (6.22)	1.06 (0.36)	0.32 (0.03)
*Consumed (Fasting Day)*												
**C**	20	574 (143)	24 (9)	1.8 (0.7)	17 (6)	78 (19)	16 (4)	27 (5)	55 (8)	7.44 (2.03)	0.32 (0.13)	0.02 (0.01)
**P**	21	598 (108)	53 (8)	4.9 (0.7)	18 (5)	54 (15)	36 (6)	27 (4)	36 (7)	7.71 (1.79)	0.67 (0.10)	0.06 (0.01)
**L**	20	511 (132)	23 (10)	8.0 (1.4)	15 (6)	63 (18)	18 (5)	25 (7)	50 (7)	6.64 (2.13)	0.30 (0.13)	0.10 (0.01)
*Consumed (Overall)*												
**C**	20	1275 (214)	51 (11)	3.7 (0.9)	41 (9)	171 (31)	16 (2)	29 (3)	54 (4)	16.46 (3.17)	0.66 (0.17)	0.05 (0.01)
**P**	21	1276 (207)	107 (14)	9.8 (1.3)	43 (10)	113 (25)	34 (4)	30 (3)	35 (5)	16.38 (3.26)	1.36 (0.16)	0.12 (0.01)
**L**	20	1175 (233)	52 (13)	16.3 (2.1)	37 (11)	141 (30)	18 (3)	28 (5)	48 (6)	15.23 (3.68)	0.68 (0.21)	0.21 (0.02)

Data presented as mean (SD). C, Control group; P, Protein group; L, Leucine group; CHO, Carbohydrates.

**Table 6:** Body composition analyses—main effect of time.

Characteristics			95% CI		
	*p*-Value	Mean Difference *	Lower	Upper	Cohen’s d
**Body mass (kg)**	<0.001	−2.2	−2.4	−1.9	−0.2
**Fat Mass (kg)**	<0.001	−1.3	−1.5	−1.0	−0.2
**Lean Mass (kg)**	<0.001	−0.9	−1.1	−0.6	−0.1
**ALM (kg)**	0.036	−0.3	−0.6	0.0	−0.1
**BMI (kg/m^2^)**	<0.001	−0.8	−0.8	−0.7	−0.2
**FMI (kg/m^2^)**	<0.001	−0.4	−0.5	−0.4	−0.2
**LMI (kg/m^2^)**	<0.001	−0.3	−0.4	−0.2	−0.2
**ALMI (kg/m^2^)**	0.047	−0.1	−0.2	0.0	−0.1
**PBF (%)**	<0.001	−0.8	−1.0	−0.5	−0.2
**Trunk Fat Mass (kg)**	<0.001	−1.1	−1.3	−0.8	−0.2
**Android Fat Mass (kg)**	<0.001	−0.2	−0.3	−0.2	−0.3
**Gynoid Fat Mass (kg)**	<0.001	−0.2	−0.3	−0.1	−0.2
**BMC (kg)**	<0.001	−0.0	−0.0	−0.0	−0.1
**BMD (g/cm^2^)**	0.274	−0.0	−0.0	0.0	−0.0

* Post minus pre values. ALM, appendicular lean mass; BMI, body mass index; FMI, fat mass index; LMI, lean mass index; ALMI, appendicular lean mass index; PBF, percentage body fat; BMC, bone mineral content; BMD, bone mineral density.

**Table 7:** Sarcopenic characteristics.

	Group	Control		Protein		Leucine	
Characteristics	Session	Pre	Post	Pre	Post	Pre	Post
	*N*	20	20	21	21	20	20
**Appendicular Lean Mass Index (kg/m^2^)**	Mean	7.9	7.8	7.9	7.8	7.9	7.8
	SD	0.6	0.8	0.7	0.8	1.0	1.1
	Min	6.8	6.9	6.9	6.7	5.9	5.8
	Max	9.2	9.9	9.3	9.0	10.2	10.6
**Handgrip Strength (kg)**	Mean	35.9	38.0	36.5	37.0	33.6	35.3
	SD	6.6	7.1	6.0	6.3	7.9	8.8
	Min	23.4	26.3	27.8	25.3	18.6	20.3
	Max	45.2	49.3	47.9	50.0	46.7	52.5
**6m Gait Speed (m/s)**	Mean	1.1	1.2	1.2	1.3	1.2	1.2
	SD	0.1	0.1	0.1	0.1	0.1	0.1
	Min	0.7	1.1	1.0	1.0	1.0	1.0
	Max	1.4	1.4	1.5	1.5	1.5	1.5
**5x Sit-to-stand (s)**	Mean	7.6	7.0	7.8	6.7	7.6	6.7
	SD	1.6	1.3	1.7	1.0	2.0	1.6
	Min	5.4	4.6	5.0	5.0	4.8	4.2
	Max	11.0	9.2	11.5	8.8	12.9	11.2
**Short Physical Performance Battery**	Mean	12.0	12.0	12.0	12.0	11.9	12.0
	SD	0.2	0.0	0.2	0.0	0.5	0.0
	Min	11.0	12.0	11.0	12.0	10.0	12.0
	Max	12.0	12.0	12.0	12.0	12.0	12.0
**Low Muscle Mass**	N (%)	1 (5)	1 (5)	1 (5)	3 (14)	2 (10)	4 (20)
**Low Muscle Strength**	N (%)	3 (15)	3 (15)	1 (5)	1 (5)	5 (25)	4 (20)
**Poor Physical Performance**	N (%)	3 (15)	0 (0)	0 (0)	0 (0)	2 (10)	2 (10)
**Sarcopenia/** **Severe Sarcopenia**	N (%)	1 (5)	1 (5)	0 (0)	0 (0)	2 (10)	3 (15)

**Table 8:** Health characteristics and cognitive function.

	Group	Control		Protein		Leucine	
Characteristics	Session	Pre	Post	Pre	Post	Pre	Post
**Systolic Blood Pressure (mmHg)**	*N*	20	20	21	21	20	20
	Mean	127	125	128	120	129	124
	SD	8	9	8	9	14	13
	Min	111	109	112	102	109	95
	Max	141	138	143	139	169	147
**Diastolic Blood Pressure (mmHg)**	*N*	20	20	21	21	20	20
	Mean	81	81	81	75	84	79
	SD	9	10	5	6	8	7
	Min	66	62	70	66	71	66
	Max	94	95	93	87	97	93
**Fasting Glucose (mmol/L)**	*N*	20	20	21	21	20	20
	Mean	5.6	5.6	5.7	5.6	5.6	5.6
	SD	0.3	0.4	0.4	0.4	0.4	0.4
	Min	4.6	5.0	4.9	4.9	5.0	5.0
	Max	6.2	6.4	6.4	6.5	6.3	6.2
**Insulin (mU/L)**	*N*	19	17	21	21	19	20
	Mean	8.5	7.7	8.8	8.4	9.4	8.1
	SD	4.9	3.4	5.2	5.6	7.1	5.0
	Min	3.4	3.3	1.4	1.1	0.5	0.9
	Max	20.0	13.1	25.7	20.3	28.6	20.4
**HOMA-IR (Homeostasis Model** **Assessment of** **Insulin Resistance)**	*N*	19	17	21	21	19	20
	Mean	2.1	1.9	2.2	2.1	2.4	2.0
	SD	1.2	0.8	1.3	1.5	1.9	1.3
	Min	0.9	0.9	0.3	0.3	0.1	0.2
	Max	4.9	3.1	6.1	5.8	7.5	5.3
**HbA1c (%, Glycated Hemoglobin)**	*N*	19	17	21	21	19	20
	Mean	5.7	5.7	5.7	5.6	5.8	5.7
	SD	0.3	0.3	0.4	0.4	0.4	0.4
	Min	4.9	4.8	4.7	4.8	4.7	4.7
	Max	6.2	6.1	6.4	6.5	6.4	6.5
**Total Cholesterol (mmol/L)**	*N*	19	17	21	21	19	20
	Mean	5.3	5.2	4.9	4.6	5.4	5.1
	SD	0.9	1.0	1.0	0.9	0.8	0.8
	Min	4.3	3.8	3.2	3.2	3.9	3.7
	Max	7.8	7.3	6.7	6.6	6.7	7.1
**HDL (High-Density Lipoprotein Cholesterol, mmol/L)**	*N*	19	17	21	21	19	20
	Mean	1.3	1.4	1.3	1.3	1.4	1.4
	SD	0.3	0.4	0.2	0.2	0.3	0.3
	Min	1.0	0.9	0.9	1.0	0.9	0.9
	Max	2.1	2.4	1.7	1.7	2.4	2.3
**Low-Density Lipoprotein Cholesterol (mmol/L)**	*N*	19	17	21	21	19	20
	Mean	3.3	3.2	2.9	2.8	3.4	3.2
	SD	0.7	0.7	1.0	0.9	0.6	0.8
	Min	2.3	2.2	1.2	1.3	2.5	1.9
	Max	5.0	4.6	5.0	5.1	4.3	5.2
**Total Cholesterol/** **HDL Ratio**	*N*	19	17	21	21	19	20
	Mean	4.2	3.9	3.8	3.7	4.0	3.8
	SD	0.9	0.7	1.1	0.9	0.8	1.1
	Min	3.0	2.9	2.2	2.4	2.7	2.3
	Max	6.3	5.5	7.2	6.8	5.7	5.8
**Triglycerides (mmol/L)**	*N*	19	17	21	21	19	20
	Mean	1.4	1.2	1.4	1.2	1.3	1.2
	SD	0.6	0.5	0.6	0.5	0.6	0.5
	Min	0.6	0.5	0.5	0.6	0.4	0.5
	Max	2.7	2.1	3.3	2.3	2.6	2.8
**MoCA (Montreal Cognitive Assessment)**	*N*	20	20	21	21	20	20
	Mean	27	27	26	27	26	27
	SD	2	2	3	2	2	2
	Min	22	24	22	21	21	21
	Max	29	30	30	30	30	30

## Data Availability

De-identified data analyzed in the manuscript are available via https://doi.org/10.25340/R4/RSF2WL.

## Abbreviations

The following abbreviations are used in this manuscript:

ADF	Alternate-day fasting
MPS	Muscle protein synthesis
NTU	Nanyang Technological University
IRB	Institutional review board
GPAQ	Global physical activity questionnaire
MET	Metabolic equivalent of task
MoCA	Montreal cognitive assessment
AWGS	Asian working group for sarcopenia
DXA	Dual-energy X-ray absorptiometry
HbA1c	Glycated hemoglobin
GEE	Generalized estimating equation
HGS	Handgrip strength
GS	Gait speed
SPPB	Short physical performance battery
5STS	Five times sit-to-stand
ALM	Appendicular lean mass
ALMI	ALM index
FM	Fat mass
FMI	FM index
PBF	Percentage body fat
BMC	Bone mineral content
BMD	Bone mineral density
RDA	Recommended dietary allowance
PA	Physical activity
